# Effectiveness of self-management of dry and wet cupping therapy for low back pain: A systematic review and meta-analysis

**DOI:** 10.1097/MD.0000000000032325

**Published:** 2022-12-23

**Authors:** Wei-Cheng Shen, Yih-Kuen Jan, Ben-Yi Liau, Quanxin Lin, Song Wang, Chien-Cheng Tai, Chi-Wen Lung

**Affiliations:** a Department of Digital Media Design, Asia University, Taichung, Taiwan; b Rehabilitation Engineering Lab, University of Illinois at Urbana-Champaign, Champaign, IL; c Kinesiology and Community Health, University of Illinois at Urbana-Champaign, Champaign, IL; d Computational Science and Engineering, University of Illinois at Urbana-Champaign, Champaign, IL; e Department of Biomedical Engineering, Hungkuang University, Taichung, Taiwan; f Department of Creative Product Design, Asia University, Taichung, Taiwan; g Division of Chinese Medicine, Asia University Hospital, Taichung, Taiwan; h International Ph.D. Program for Cell Therapy and Regeneration Medicine, Taipei Medical University, Taipei, Taiwan.

**Keywords:** Oswestry disability index, pain intensity, present pain intensity, quality of life, Vsisual Analogue Scale

## Abstract

Low back pain (LBP) can significantly affect a person’s quality of life. Cupping has been used to treat LBP. However, various cupping methods are typically included in evaluating the efficacy of cupping therapy. Therefore, the objectives of this study were to evaluate the evidence from the literature regarding the effects of dry and wet cupping therapy on LBP in adults. Dry and wet cupping therapy are analyzed categorically in this study.

**Methods:**

We searched for randomized clinical trials with cupping in LBP published between 2008 and 2022. In dry or wet cupping clinical studies, pain intensity was assessed using the Visual Analogue Scale and present pain intensity, and the quality of life intensity was measured using the Oswestry disability index.

**Results:**

The 656 studies were identified, of which 10 studies for 690 patients with LBP were included in the meta-analysis. There was a significant reduction in the pain intensity score with present pain intensity using wet cupping therapy (*P* < .01). In addition, both cupping therapy groups displayed significant Oswestry disability index score reduction compared to the control group (both *P* < .01). The patients with LBP have a substantial reduction by using wet cupping but have not shown a considerable decrease by using dry cupping (*P* = .19). In addition, only wet cupping therapy groups displayed a significantly improved quality of life compared to the control group. The study had a very high heterogeneity (*I*^2^ > 50%). It means there is no standardization in the treatment protocol in randomized clinical trials. In the meta-regression, there was statistically significant evidence that the number of treatment times and intercepts were related (*P* < .01).

**Conclusion:**

The present meta-analysis shows that wet cupping therapy effectively reduces the pain intensity of LBP. Furthermore, both dry wet cupping therapy improved patients with LBP quality of life.

**Strengths and limitations of this study**

- Various cupping methods are usually included in the efficacy analysis of cupping therapy.
- The originality of this study lies in the categorical meta-analysis of the benefits of dry and wet cupping.
- We further found that the scope of application of cupping methods in LBP differed based on the existing cupping data.
- This study is limited due to the lack of studies comparing wet cupping with dry cupping, so it can only analyze the impact based on quantitative analyses.

## 1. Introduction

Low back pain (LBP) is a significant global problem.^[1]^ The 70% of the general adult population suffers from LBP at least once in their lifetime, and most people with LBP have restricted activities of daily living.^[2]^ The LBP is among the costliest, with an estimated $134.5 billion paid across private (57%), public (34%), and out-of-pocket payers in the United States.^[3]^ LBP frequently results in significant impairment or decline in the performance of social responsibilities, including work and family.^[4]^ LBP is a potentially harmful impact on quality of life, such as limitations on body activity or condition of psychosocial health.^[4]^

In general, the first LBP occurs within 30 years of age, and the prevalence increases until age 65 to reach its peak and then decreases with advancing age.^[5]^ There is a high rate of recurrence of LBP, approximately 30% of people who recover from LBP experience recurrence within 1 year.^[6]^ Most patients with LBP who experience an acute episode of LBP recover within 2 to 4 weeks,^[7]^ but 20% of acute LBP will continue experiencing symptoms beyond 3 months and become chronic patients with LBP.^[2]^ Three common treatments in LBP are pharmaceutical therapy, surgical procedures, or rehabilitation.^[8]^ As the first-line treatment, pharmacologic therapy is the most widely used treatment in clinical practice.^[9]^ It often involves acetaminophen and non-steroidal anti-inflammatory drugs,^[10]^ which could provide reliable pain relief for patients with LBP.^[9]^ However, pharmacologic therapy is not recommended for chronic pain because it can damage liver and provoke hemorrhagic gastritis.^[11]^ For the second-line treatment, physiotherapy and exercises are advocated as effective treatments for chronic LBP.^[8]^ There is evidence of effective treatment for chronic pain by therapy over 12 months.^[9]^ However, rehabilitation is not routinely or widely available to patients with chronic pain of the LBP because rehabilitation programs require finance, space, and clinician-involved training.^[12]^ In the third-line treatment, surgery is reliable when the pain is not tolerable.^[9]^ However, surgical options are costly and carry a greater risk to the patient with LBP.^[13]^ The option should only be considered after medication and physical therapy have failed.^[14]^ As a result of the side effects of current conventional therapies, complementary and alternative medicine (CAM) has grown in popularity over the past decade in developed countries.^[15]^ CAM has been investigated for its clinical effectiveness in LBP in many studies.^[16]^ Several CAM treatments are effective in managing LBP, even though they may also have side effects.^[17]^

Cupping therapy is a centuries-old method to relieve and improve LBP.^[18]^ The reliability of cupping therapy as one of the treatments in CAM has gained wide acceptance internationally.^[8]^ Furthermore, many studies have shown an interest in cupping therapy for treating LBP because they believe cupping therapy is considered safe and productive. Some scholars found that cupping therapy can reduce pain,^[16,19,20]^ and improve quality of life.^[21–23]^

Even though the mechanism of action of cupping therapy remains unclear, it has been reported that it involves effects on pain, including neural, hematological, immune, and psychological consequences,^[24]^ or stimulation of the skin causes several autonomous, hormonal, and immune reactions.^[25]^ However, cupping therapy outcomes does not always fulfill the expectation of therapists and patients with LBP.^[26]^ Some scholars found that treatments don’t work for reducing pain^[19]^ or improving quality of life.^[23]^ The cupping even increases in despair of the muscle soreness.^[27]^

Numerous clinical trials and randomized clinical trials (RCTs) studies have recently emerged for clinical effectiveness. Unfortunately, their conclusions are far from uniform.^[28]^ Conflicting decisions about cupping regarding pain treatment outcomes could be related to the differences in cupping therapy practice, which employ different cupping therapy forms and give different results, and are often lumped together at RCTs.^[26,29]^

Contradictory conclusions may be found from cupping therapy practice.^[30,31]^ As we know, the classification of cupping therapy practice is categorized broadly into dry and wet cupping. Dry cupping is a technique in which cups are applied to the skin to create a vacuum for suction without drawing blood. Wet cupping contains 2 steps: before suctioning the cups, practitioners make small incisions with a triangle-edged needle or plum-blossom needle, firmly tapping the acupoint for a short time to cause bleeding,^[32]^ and then creating a vacuum for suctioning the skin. It may result in a different effect between dry and wet cupping because bloodletting also carries a remarkable potential for various disorders and pain reduction.^[33]^

Since pain and physical disability are subjective feelings, self-management to quantify them is needed.^[22,29]^ Therefore, self-management is a favorable option for symptom management for patients with LBP, which can evoke their consciousness and enthusiasm for individual responsibility for their health.^[34]^

The evaluation of recent clinical studies demonstrated that cupping therapy is an effective modality for pain treatment.^[35]^ However, scholars have proposed many theories to explain the effects of cupping, including pain gate theory, diffusion toxicity inhibitory control, reflex zone theory, nitric oxide release theory, immune system activation theory, etc.^[36,37]^ However, none of these opinions have been proven by scientific studies stimulating the peripheral nervous system by draining extra fluids and moving connective tissue.^[35]^ Therefore, we can still use self-management (Visual Analogue Scale [VAS], present pain intensity [PPI], and Oswestry disability index [ODI]) for evaluating body information outcomes.^[38]^

The VAS is the most widely used as it is simple and requires little assessment time to assess the pain,^[39]^ And it is one of the most frequently used scales for LBP.^[34]^ Another of the best-known scales for pain is the PPI, a questionnaire used to measure pain intensity.^[40]^ In research, PPI has become increasingly recognized to measure the LBP level as it can be obtained quickly.^[41]^ And ODI is recommended for use when measuring the impact of LBP on quality of life because it is designed to reflect a disability index for LBP.^[42]^

Cupping is often used as a symptomatic treatment for a wide range of conditions in clinical pain. Currently, dry and wet cupping therapies are often used as the same symptomatic treatment discussion in LBP.^[43]^ However, many clinicians are skeptical about its value because its clinical effectiveness remains uncertain.^[30]^

We believe the dry and wet cupping classifications have due to the different cupping procedure methods.^[30]^ However, until now, the meta-analyses have not clarified the effect of dry and wet cupping.^[26,44,45]^ In most studies, cupping therapy was compared with prescriptive care, but there is no classification of cupping therapies. Therefore, we use the classification of dry and wet cupping to clarify what kind of cupping can reduce pain and improve quality of life.

Therefore, it may overlook the effect differences between dry and wet cupping. This study analyzes dry and wet cupping separately in the meta-analyze by self-management, aimed to assess current RCTs of cupping therapy for LBP to examine pain and quality of life at both cuppings.

The originality of our study is that we analyze the effectiveness of wet cupping and dry cupping samples for the first time in the studies on LBP by self-management.

## 2. Method

### 2.1. Search strategy

The search was conducted in August 2022, and citations were uploaded to the Covidence online software. Our systematic review protocol is registered with PROSPERO (registration number: CRD42022354704). The study was based on the criteria of the Preferred Reporting Items for Systematic Reviews and Meta-analyses (PRISMA Statement).^[46]^

Two investigators (W.-C.S. and C.-W.L.) will execute the structured and systemic literature retrieval without interfering with each other in the following 5 electronic bibliographic databases: PubMed, Web of Science, Cochrane Library, Scopus, and CINAHL. Interrater agreement was assessed using Cohen’s κ (κ = 0.949). Discrepancies were resolved through discussion. The search period will be from January 2008 to December 2021 in our study. We will appropriately adjust the search strategy according to the different databases in our research. Details of the search strategy are presented in Appendix 1, Supplemental Digital Content, http://links.lww.com/MD/I148.

Subsequently, they independently conducted the full-text review and jointly decided on the final pool of articles included in the study. Reference list searches and cited references were conducted based on the full-text articles meeting the study selection criteria identified from the keyword search. Articles identified were further screened and evaluated using the same study selection criteria. Reference searches repeated new-identified articles until they found no additional relevant article.

### 2.2. Study selection criteria

This study included articles published up to 2022 as new clinical studies investigating cupping. Studies included in this review met all of the following criteria: the study was written in English and published until July 2022; the subjects included were male or female, subjects are over 18 years of age with LBP of any duration; at least one of the treatments evaluated was related to cupping; the RCTs at least evaluated one of the VAS, PPI, or ODI; and follow-up should be shown after the end of all treatments outcome measures or reports at more time checkpoints.

Studies were excluded from the review if they met one or more of the following criteria: the dissimilarity in the basal measures between groups could falsely contribute to the pooled effect; observational studies or nonpeer-reviewed articles such as dissertation or conference proceeding; and studies that looked at cupping therapy combined with other traditional Chinese medicine, such as acupuncture, compared with non-CAM therapies.

### 2.3. Self-management scale

Self-management is a strategy people use to quantify physiological pain and its impact.^[47]^ Self-management for LBP is widespread due to the essential of quantifying data to prove the efficacy of interventions.^[48]^

VAS and PPI are the self-management scale recommended for assessing the pain for LBP,^[39,40]^ and ODI is the self-management scale recommended for the disability index for LBP.^[42]^

The VAS scores are a scale with ten numbers and elucidated as follows: no pain (0), mild (1–3), moderate pain (4–6), severe pain (7–9), and the worst pain possible (10), and is frequently used in patients with chronic musculoskeletal pain.^[29]^

PPI is a qualitative assessment that presents 6 words that describe the subject’s experience: no pain, mild, discomforting, distressing, horrible, and excruciating. Each participant was instructed to point out the one that best described her pain at the time of the interview.^[22]^

ODI is a scale developed for assessing the loss of living function in LBP. It includes ten questions on the limitations of activities of daily living. Each item is rated on a 0 to 5 point scale and transferred into a percentage score.^[49]^

### 2.4. Data extraction

This study used a standardized data extraction form to collect the following methodological and outcome variables from each included study: article identification (title, author, journal, year of publication, country, language of the survey); clinical data (number of patients with LBP by gender, mean age, diagnosis, duration of symptoms); objectives; and methodological characteristics (design, sample size and loss to follow-up; inclusion and exclusion criteria). Description of the interventions in the follow-up group: number of treatments, duration of treatment, type of technique applied (dry cupping and wet cupping), the device used, device dwell time, suction method (automatic/manual fire), and the results evaluation methods: evaluation times, evaluation interval, measurement tools, data analysis, main results, and research results.

All the information data were extracted and transferred into Excel to calculate frequency. Data were summarized using a risk ratio with 95% confidence intervals (CIs) for binary outcomes or mean difference with a 95% CI for continuous products. RevMan software version 5.3 (Cochrane Library Software, Oxford, UK) was used for data analyses in appraising the included RCTs. Meta-analysis was used in the studies and had an excellent homogeneity in study design, participants, interventions, control, and outcome measures.

This study adapted form based on the recommendations of the standards for reporting interventions in clinical studies of cupping (STRICTOC) to collect information on the selected research and the classification of cupping therapy.

### 2.5. Statistical analysis

We conducted data analyses using the selected standard mean difference with 95% CI to describe the mean differences between the cupping group and the control group or dry cupping and the wet control group. A *P* value less than .05 was judged as statistically significant.

Statistical heterogeneity was assessed by the *I*^2^ index.^[50]^ The level of heterogeneity represented by the I^2^ index was interpreted as small (*I*^2^ ≤ 25%), moderate (25% < *I*^2^ ≤ 50%), large (50% < *I*^2^ ≤ 75%), or very large (*I*^2^ > 75%). A fixed-effect model would be estimated when a small or moderate heterogeneity was present. A random-effect model would be estimated when extensive heterogeneity was present (Stata Corp LP; College Station, TX, USA).^[51]^ Through evaluative meta-regression, we attempted to identify factors that contribute to high heterogeneity.

### 2.6. Study quality assessment

The RCTs’ quality was evaluated using a biased scale comprising 10 items,^[52]^ including random sequence, allocation concealment, blinding, incomplete outcome data, selective reporting, and other biases.

## 3. Results

### 3.1. Study selection

**Figure 1.**
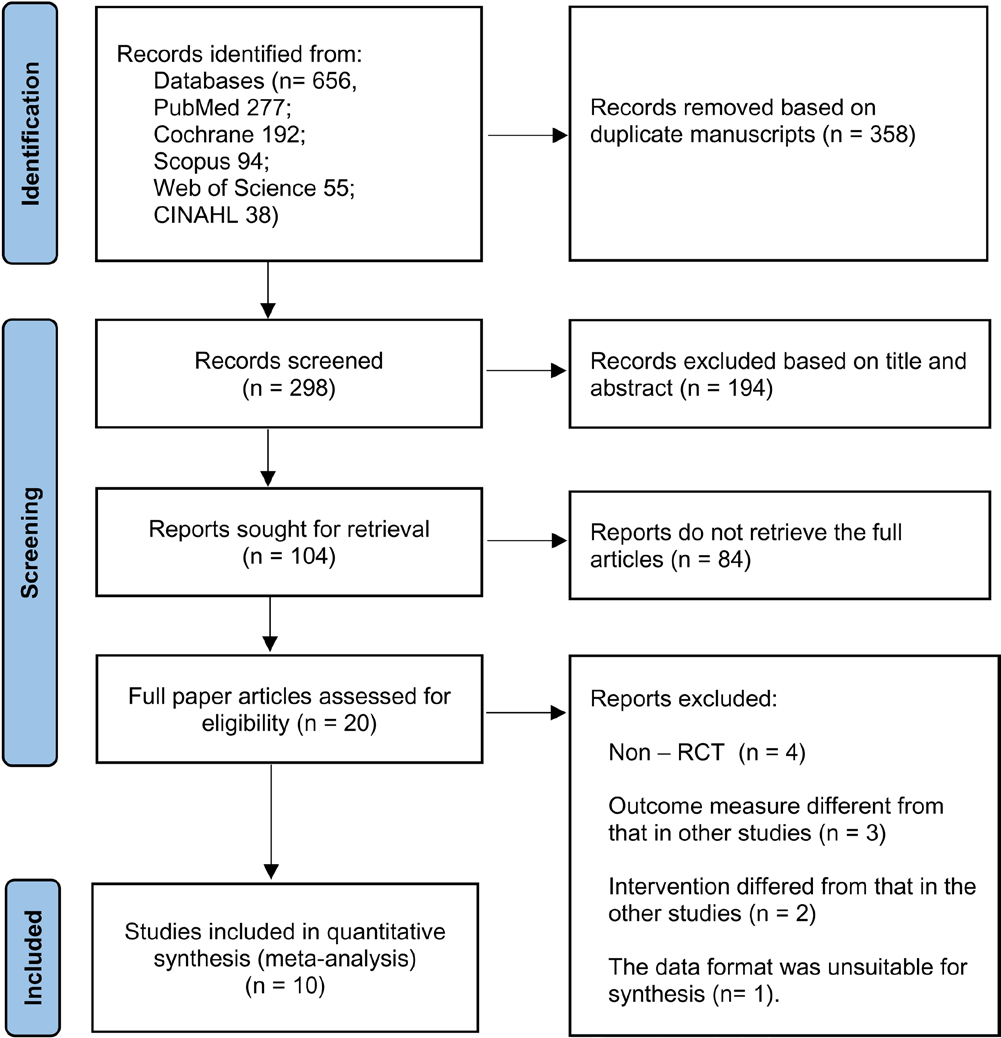
Flowchart of study selection.

The literature search of databases generated 690 articles. As a result of excluding the 358 duplicate manuscripts articles, 194 ineligible articles by reviewing the title and abstract, and 84 articles that did not retrieve the full text articles, we analyzed 20 full-text articles. For the systematic review, 10 articles were excluded as they did not meet the inclusion criteria. Following the full-text review process, 10 eligible RCTs with 690 participants were included (Fig. 1).

**Figure 2.**
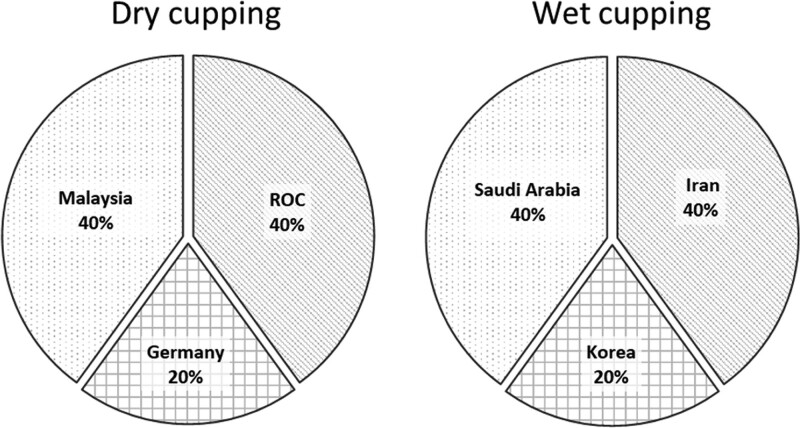
Characteristics of selected studies.

Among the included 10 studies, wet cupping was tested in Iran,^[35,53]^ Korea,^[54]^ and Saudi Arabia.^[21,37]^ Dry cupping was tested in Republic of China,^[19,20]^ Germany,^[16]^ and Malaysia^[23,55]^ summarized in Figure 2, 4 studies reported the effect of cupping on VAS,^[16,19,20,35]^ 4 studies reported PPI,^[21,37,53,54]^ and 7 studies reported ODI.^[21,23,35,37,53,54,55]^

The number of participants in each study ranged from 13 to 90 people. All participants were adults with LBP.

### 3.2. Description of interventions (Characteristics of selected studies)

As Table 1, On dry cupping, 3 studies provided results from the VAS^[16,19,20]^; 2 from the ODI^[23,55]^; and none from the PPI results. On the wet cupping, one study provided results from the VAS^[35]^; 5 studies provided results from the ODI^[21,35,37,53,54]^; and 4 studies provided results from the PPI.^[21,37,53,56]^ The results showed that cupping therapy offers advantages in alleviating quality of life over the usual care for patients with LBP.

**Table 1:** Included studies with self-management effects.

	Self-management	Group 1	Group 2
Three studies	VAS	Dry cupping	Regular intervention
Four studies	PPI	Wet cupping	Regular intervention
Two studies	ODI	Dry cupping	Regular intervention
Four studies	ODI	Wet cupping	Regular intervention

ODI = Oswestry disability index, PPI = present pain intensity, VAS = Visual Analogue Scale.

Table 2 shows the characteristics of the studies regarding the objectives, the interventions applied in the experimental and control groups, and the primary study outcomes. A total of 300 subjects participated in the dry cupping studies, of whom 151 subjects were in the experimental treatment group and 149 subjects in the control group (sham treatment, waiting list, standard medical/active treatment, or no treatment). The selected studies were cases of cupping for LBP. The 5 studies’ interventions were mainly provided by physicians.^[16,19–21,53]^ One study was followed by nurses.^[16]^ Three studies were followed by therapists.^[23,35,54]^ One studies^[23]^ reported that therapists provided the intervention but did not specify the training area. Three studies did not establish the operator’s identity.^[37,55]^

**Table 2:** Characterization of the studies regarding the applied intervention.

Study	Objective	Intervention in the experimental group	Intervention in the control group	Main findings	Outcomes	Self-measurement tools	Interval
AlBedah et al 2015^21]^	To evaluate the effectiveness and safety of wet cupping as a treatment for persistent and nonspecific low back pain	Wet cupping analgesics (n = 40)	Analgesics (n = 40)	For at least 2 wk after the end of the intervention, wet cupping can reduce pain and improve disability associated with nonspecific and persistent low back pain	Pain intensity Physical disability	Numeric scale (0–100) PPI ODI	Baseline, after a follow-up of 2 wk
Kim et al 2011^[54]^	To determine the safety and efficacy of wet cupping in the treatment of intractable nonspecific low back pain	Wet cupping (n = 21)	Usual care in both groups (n = 11)	Wet cupping may be potent and reduce crated with nonspecific and persistent low back pain	Pain intensity Physical disability	Numeric scale (0–100) PPI ODI	Baseline, after a follow-up of 2 wk
Farhadi et al 2009^[53]^	To evaluate the effectiveness of wet cupping in treating intractable and nonspecific low back pain	Wet cupping (n = 48)	Usual care(n = 50)	Wet cupping offers more excellent short-term clinical benefits than conventional cars	Pain intensity Physical disability Medication use	PPI ODI Medication Quantification Scale	Baseline and after 3 mo of follow-up
Mardani-Kivi et al 2019^[35]^	To compare the possible effects of wet-cupping therapy with conventional therapy on persistent nonspecific low back pain	Wet cupping (n = 84)	Usual care(n = 83)	Wet cupping may be a proper method to decrease persistent nonspecific low back pain without conventional treatment	Pain intensity Physical disability	Vas (0–10) ODI	A total of 2 treatments for 4 wk, assessments in the first month, the second month, and the sixth month
Lin et al, 2012^[19]^	To evaluate the effects of laser acupuncture and cupping on low back pain	Laser acupuncture and dry cupping (n = 28)	False dry cupping and radiation-free laser (n = 29)	Laser acupuncture and gentle cupping therapy may be appropriate treatments for patients with low back pain	Pain intensity Physical disability	VAS (0–10)	Assessments for 5 consecutive days - 2 before and two after
Teut et al 2018^[16]^	To study the effectiveness of dry cupping in reducing pain and improving back function and quality of life in patients with chronic nonspecific low back pain	Intense negative pressure dry cupping with paracetamol on demand (n = 37) Weak negative pressure pulsed cupping with paracetamol on the market (minimal cupping) (n = 36)	Paracetamol as needed (maximum dose is 4 doses of 500 mg/d) (n = 37)	Both types of cupping were effective for nonspecific low back pain, with no significant differences in direct comparisons after 4 wk. However, only dry cupping showed an effect after 12 wk compared to the control group	Pain intensity Quality of life	VAS (0–10) Ryodoraku	Assessments for 5 consecutive days - 2 before and two after
Lin et al, 2017^[20]^	To illustrate the effectiveness of acupuncture and Chinese cupping therapy in treating lower back pain	Laser acupuncture and dry cupping (n = 25)	Sham Laser and dry cupping (n = 23)	Laser acupuncture combined with TCM cupping therapy can effectively reduce back pain. In addition, changes in plasma cortisol levels suggest that laser acupuncture combined with TCM cupping therapy is an effective pain relief therapy	Pain intensity Physical disability	ODI	5, 10, 15, and 20 min as well as 24 h after treatment
Silva et al, 2021^[55]^	To evaluate the effects of dry cupping on pain intensity	Dry cupping (n = 45)	Sham dry cupping (n = 45)	Dry cupping therapy was not superior to sham cupping for improving pain, physical function, mobility, quality of life, psychological symptoms, or medication use in people with nonspecific chronic low back pain	Pain intensity	SF-36#	Once a week for 8 wk
Razali and Choo 2021^[23]^	To identify the effectiveness of dry cupping and hot pack on pain relief and reduce functional disability for patients with nonspecific low back pain	Dry cupping (n = 13)	No treatment (n = 13)	Both dry cupping and hot pack were effective interventions for pain relief and reduced functional disability among patients with nonspecific low back pain	Pain intensity Physical disability	NPR ODI	Baseline, after a follow-up of 3 wk
Al-Eidi et al, 2019^[37]^	To evaluate the feasibility of comparing the effect of the traditional Hijamah and the Asian wet cupping techniques in managing patients with chronic low back pain	Asian cupping group (n = 35)	Traditional cupping group (n = 33)	There is no significant difference between the 7 d and the 14th day after the test	Pain intensity Quality of life	PPI ODI	Baseline, after a follow-up of 2 wk

ODI = Oswestry disability index, PPI = McGill Present Pain Intensity questionnaire, SF-36 = Short Form 36 Health Survey Questionnaire, TCM = Traditional Chinese medicine, VAS = Visual Analogue Scale.

The selected studies were all cases of cupping for LBP. The most evaluated outcome was 8 studies on pain intensity,^[16,19–21,35,37,53,54]^ followed by 6 studies on physical disability.^[21,23,35,53,54,55]^ Table 3 shows the intervention protocol’s characteristics based on the STRICTOC classification. The 10 RCTs mention the cupping by a tank. Two RCTs used automatic cupping machines.^[16,53]^ Only one experiment used the distance pulled the skin up in the tank to measure negative pressure,^[20]^ the 4 RCTs used manual suction pumps,^[21,28,35,37]^ and the last one RCTs did not specify equipment for cupping.^[19]^ Eight studies compared the effects of dry and wet cupping on reducing physical disability. The studies report that within-group in ODI changes were included in the meta-analysis and performed a pooled analysis of differences in ODI changes.

**Table 3:** The characteristics of the studies regarding the objectives, the interventions applied in the experimental and control groups, and the primary study outcomes.

Study	Total treatment number of times	Duration of treatment	Application device	Time of stay of the device	Suction method/suction strength	Peculiarities of the intervention	Application points
AlBedah et al 2015^[21]^	6	2 wk	Disposable 40 cc cups	5 min	Manual (suction pump)	Disposable lancets perforated the skin at 6 points along with the marked site 2 mm deep	Two acupoints between BL23, 24, and 25 (in each session, practitioners chose the two most painful points when pressed manually. When there were no pain points, and chose the bilateral B25)
Kim et al 2011^[54]^	6	2 wk	Disposable 40 cc cups	5 min	Manual (suction pump)	Disposable lancets perforated the skin at 6 points along with the marked site 2 mm deep	Two acupoints (the most painful when manually pressed or when there were no pain points, we chose bilateral BL25)
Farhadi et al 2009^[53]^	3	1 wk	Plastic cups – the cup size was based on the size of the patient’s body (75 or 120 cc)	3–5 min for the dry suction cup and then another 3–5 min for the wet suction cup	Automatic/manual (electric suction or, due to technical reasons, manual suction)	Surface incisions were made on the skin using the “Multiple superficial incisions” technique with 15–21 size sterile surgical slides	(A) between the 2 scapulae, opposite to the scapular spine, at the level of the thoracic vertebrae 1-3, in Phase 1; (B) the area of the sacrum, between the lumbar vertebra and the coccyx bone, in Phase 2; and (C) the calf area on the middle surface of the gastrocnemius the muscle in Phase 3
Mardani-Kivi et al 2019^[35]^	4	4 wk	Wet cupping	75–120 mm cupping cups, each treatment is performed five times, each time 20 min	Manual (suction pump)	No special narrative	On the inter-scapular area around the T2–T4 on day 1; on the sacrum area, between the lower vertebrae and the coccyx
Lin et al, 2012^[19]^	1	10 min	Laser LA400 (United Integrated Services Co, Ltd., Taiwan)/It does not describe the suction cup material	10 min	Soft cupping	No special narrative	BL40 Ashi Points
Teut et al 2018^[16]^	8	4 wk	Silicone cup	8 min	Automatic pumping	No special narrative	Point in the lumbar region
Lin et al, 2017^[20]^	1	15 min	Approximately 300 mm Hg twice aspirated	15 min	300 mm Hg	No special narrative	BL22, BL23, BL24, and BL25
Silva et al, 2021^[55]^	1	15 min	Samora cups	15 min	300 millibars (225 mm Hg)	Two suctions in the manual suction pump	BL22, BL23, BL24, BL25, and BL26
Razali and Choo 2021^[23]^	5	1 wk	Laser/6 cm diameter cups	5 min	The suction of each cup was applied until the skin rose to 1 cm	The physician administered the treatment to all patients with LBP between 3 and 6 hours (time of exuberant flow of the bladder meridian)	Muscles of the lower back at the level of the spinal discs 2–5
Al-Eidi, et al, 2019^[37]^	3	2 wk	Disposable plastic cups of 40 cc	5 min	Manual (suction pump)	Puncture (using auto-lancet) into 2 mm depth.	BL23, BL24, and BL25

BL22–26 = the acupuncture points of lumbar vertebrae in the proximal area.

None of the studies on LBP used dry cupping and wet cupping at the same experience. LBP levels were evaluated using 3 scales in this study. In the first scale, VAS was used to measure mean pain intensity, the second was PPI for current pain intensity, and the third was ODI for disability of life. Regarding the RCTs’ methodological quality were showed in Table 4, the included RCTs scored moderately on the Physiotherapy Evidence Database scale, with a mean and standard deviation of 6 ± 2.3.

**Table 4:** Score for the included studies according to the physiotherapy evidence-based database scale.

Study	Random allocation	Concealed allocation	Similarity at baseline	Subject blinding	Therapist blinding	Assessor blinding	Dropout, % (score)	Intention-to-treat analyze	Between group comparison	Point and variability measures	Total score
AlBedah et al 2015^[21]^	1	0	1	0	0	0	1	0	1	1	5
Kim et al 2011^[54]^	1	1	1	1	1	1	1	1	1	1	10
Farhadi et al 2009^[53]^	1	0	0	0	0	0	0	1	1	1	4
Mardani-Kivi et al 2019^[35]^	1	0	0	0	0	0	1	0	1	1	4
Lin et al, 2012^[19]^	1	1	1	1	1	1	1	0	1	1	9
Teut et al 2018^[16]^	1	0	0	0	0	0	0	0	1	1	4
Lin et al, 2017^[20]^	1	0	0	0	0	0	0	1	1	1	4
Silva et al, 2021^[55]^	1	1	0	1	0	0	1	1	1	1	7
Razali and Choo 2021^[23]^	1	1	1	0	1	0	0	1	1	1	8
Al-Eidi et al, 2019^[37]^	1	0	1	0	0	0	0	1	1	1	5

The means and standard deviation of total score: 6 ± 2.3. Items are scored as present (1) or absent (0), and a total score is obtained by summation. For example, the item “dropout” is scored 1 if the dropout rate is <15%.

**Figure 3.**
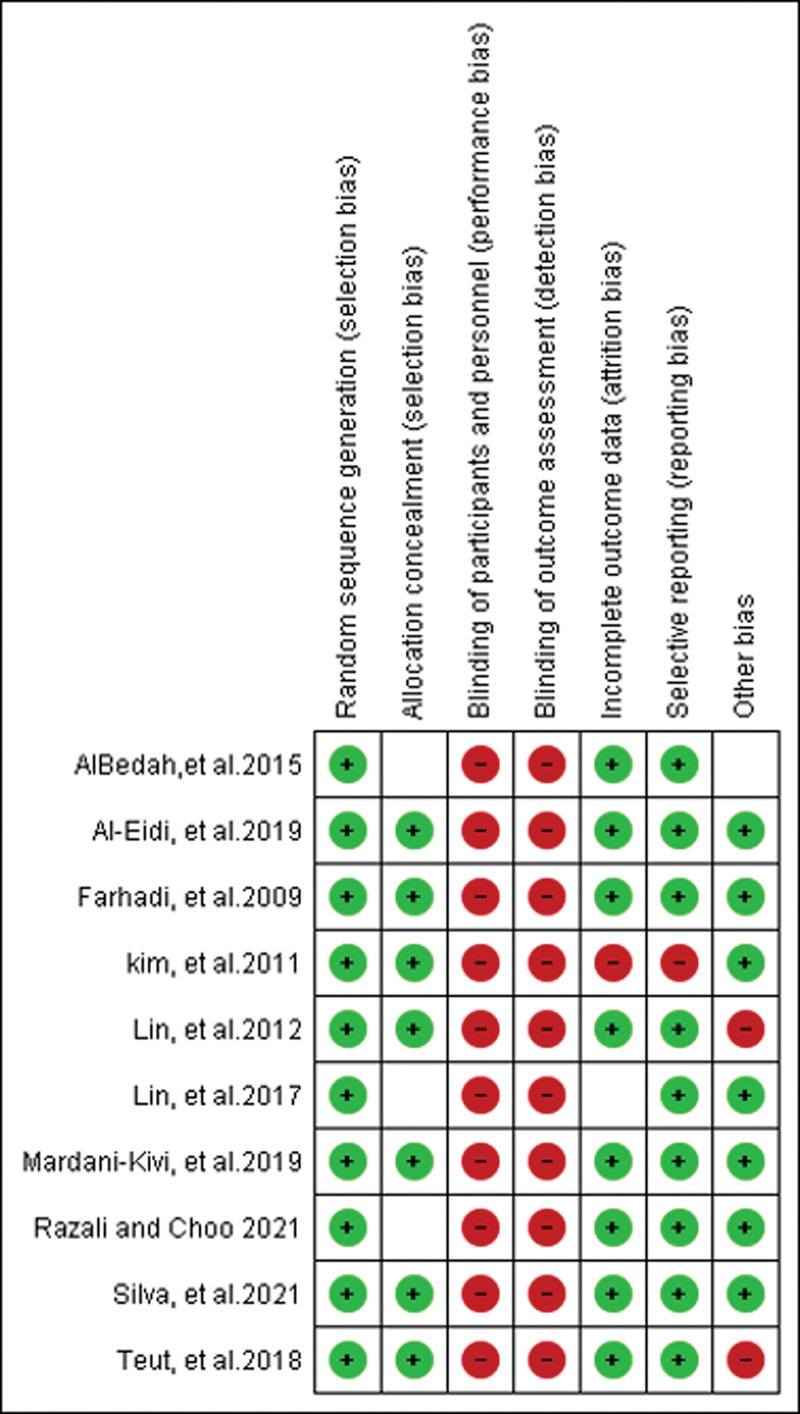
Risk of bias summary: review authors’ judgments about each risk of bias item for each included study.

**Figure 4.**
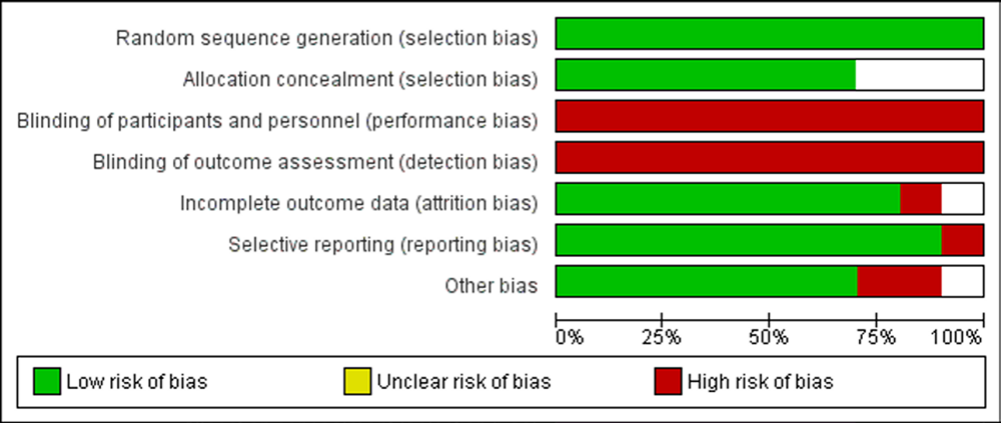
Risk of bias graph: review authors’ judgments about each risk of bias item presented as percentages across all included studies.

Additionally, Figures 3 and 4 provides information regarding the bias of RCTs. We assessed the methodological quality of RCTs using the risk-of-bias assessment tool described in the Cochrane Handbook. For random sequence generation, a low risk of bias was given to all of the included studies. For allocation concealment, most studies used the participants sequentially numbered opaque envelopes, the randomization sequence generation treatments group, or the control group. The group assignment was adequately concealed in 70% of included trials. The rest of the trials were given an unclear risk of bias. For the attrition bias, only 20% of included trials adequately reported incomplete outcome data, which may lead to attrition bias. Finally, subject blinding is difficult for cupping therapy. Although part of the reference described single blinding by fake cupping, the marks left by the suction cups are often visible in cupping therapy. Those marks may persist for several days, making it difficult to perform a masking process. Hence, all studies were considered not to have blinded their investigators and participants. Assessor blinding is possible. Unfortunately, none of the RCTs included in the systematic review adopted assessor blinding, which may result in detection biases.

### 3.3. Effect of dry cupping on VAS

Dry cupping did not show significantly reduced VAS scores more to regular interventions. Two studies examined the effect of the cupping group on VAS. And the meta-analysis of VAS did not include wet cupping in the study because we only had one RCTs on wet cupping.^[35]^ Therefore, VAS did not include wet cupping variables to determine whether dry cupping effectively treats LBP.

**Figure 5.**
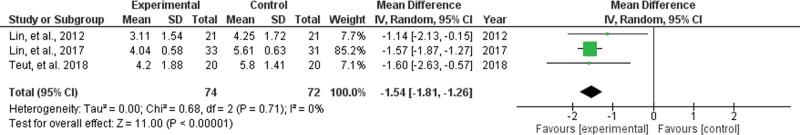
Forest plot of the mean of dry cupping in VAS. The squares represent the mean difference for each study, while the rhombus represents the aggregated average of the mean differences. control = non-cupping group, df = degrees of freedom, experimental = wet cupping group, IV = inverse variance, VAS = Visual Analogue Scale.

The interventions were combined to produce a summary statistic, the mean difference. The cupping group reported significantly lower VAS in these studies than the control group after the intervention. Estimates from the meta-analysis showed that VAS was considerably lower in the post-intervention transport cupping group than in the control group, with a mean of −1.54 (95% CI = −1.81 to −1.26, *P* = .71; *I*^2^ = 0%) (Fig. 5).

### 3.4. Effect of dry and wet cupping on PPI

Wet cupping reduced PPI scores more than regular interventions. Four studies provided evidence of the effect of wet cupping on PPI from RCTs. Among them, the average value of each result of the cupping group on PPI was lower than the control group. Therefore, the difference in PPI between the cupping group after the intervention and the control group was linked to obtaining summary statistics, the mean difference.

**Figure 6.**
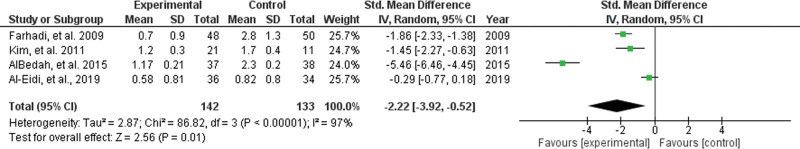
Forest plot of the mean of wet cupping in PPI. The squares represent the mean difference for each study, while the rhombus represents the aggregated average of the mean differences. control = non-cupping group, df = degrees of freedom, experimental = wet cupping group, IV = inverse variance, PPI = present pain intensity.

Significantly lower PPI in the cupping group than in the control group was reported in these studies after the intervention. Estimates from the meta-analysis showed that PPI was considerably lower in the post-intervention transport cupping group than in the control group, with a mean of −2.22 (95% CI = −3.92 to −0.52, *P *< .01; *I*^2^ = 97%) (Fig. 6).

### 3.5. Effect of dry cupping on ODI

Dry cupping reduced ODI scores more than regular interventions. Among the 5 studies about the wet cupping effect on ODI, one of the wet cupping groups came close in the experiment group than in the control group,^[53]^ and the other experiments had higher means in the control group than in the cupping group. Thus, after the intervention combined the differences in ODI Between the wet cupping groups and control groups with the mean difference, produce a summary statistic, the mean difference.

**Figure 7.**
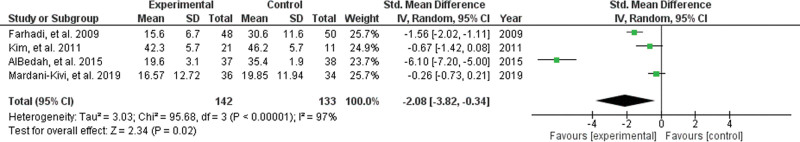
Forest plot of the mean of wet cupping in ODI. The squares represent the mean difference for each study, while the rhombus represents the aggregated average of the mean differences. control = non-cupping group, df = degrees of freedom, experimental = wet cupping group, IV = inverse variance, ODI = Oswestry disability index.

In the post-intervention cupping group, there was significantly lower ODI in these studies compared to the control group, with a mean of −2.08 (95% CI = −3.82 to −0.34, *P* < .01; *I*^2^ = 99%) (Fig. 7).

### 3.6. Effect of wet cupping on ODI

**Figure 8.**
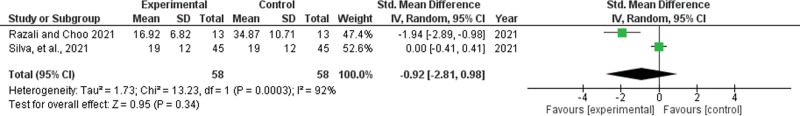
Forest plot of the mean of dry cupping in ODI. The squares represent the mean difference for each study, while the rhombus represents the aggregated average of the mean differences. control = non-cupping group, df = degrees of freedom, experimental = wet cupping group, IV = inverse variance, ODI = Oswestry disability index.

Dry cupping does not reduce ODI scores more than regular interventions. Therefore, we calculated a summary statistic based on the interventions and the mean difference. Estimates from the meta-analysis showed that the dry cupping group’s post-intervention ODI had no sign does not significantly different from the control group. The mean difference is −2.08 (95% CI = −3.82 to −0.34, *P *< .01; *I*^2^ = 99%) (Fig. 8).

### 3.7. Meta-regression

Meta-regression analysis investigated the potential effects of clinical confounders, including numbers of treatment times, dosage, and treatment duration to treatment effect.

#### 3.7.1. Number of treatment times.

**Figure 9.**
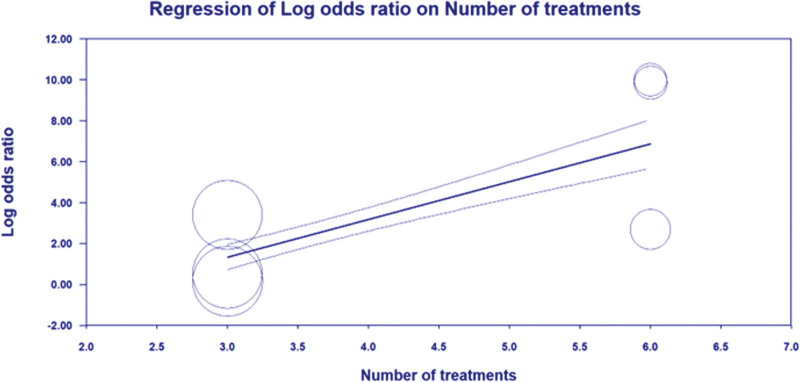
Meta-regression bubble plot of correlation between log odds ratio of PPI and the number of treatment times. PPI = present pain intensity.

**Figure 10.**
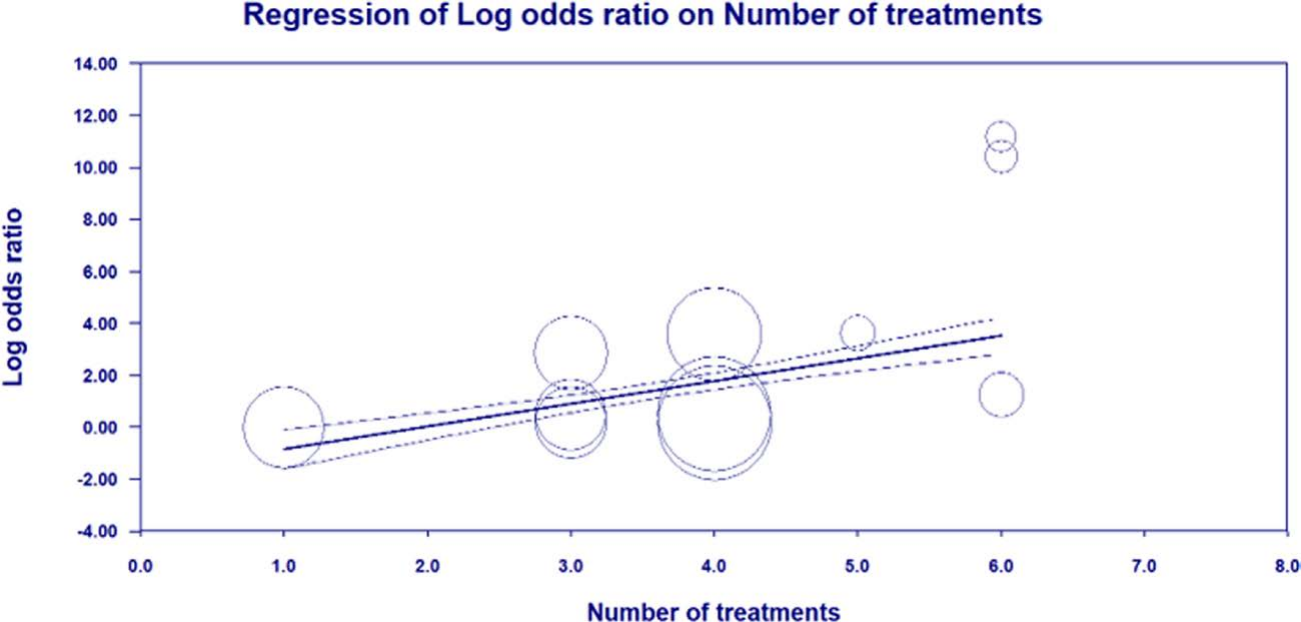
Meta-regression bubble plot of correlation between log odds ratio of ODI and the number of treatment times. ODI = Oswestry disability index.

Random-effects meta-regression revealed statistically significant evidence for an association between the log odds ratios for PPI and ODI scales intercept and numbers of treatment times (*P* < .05) (Figs. 9 and 10). This underlines that the PPI and ODI scale has reduced over more times treatment and that there is a significant association. The slope coefficient, standard error, and *P* value were as follows: PPI scale (slope coefficient = 2.0302 (0.7017), *P* = .0038), ODI scale (slope coefficient = 1.6376 (0.4498), *P* = .0003).

#### 3.7.2. Duration of treatment.

Duration of treatment was available in 4 studies (availability of information: 145/280 patients, 51.8%). Meta-regression showed no statistically significant association between the duration of treatment and the PPI scale (slope coefficient = −0.0199 (0.4774), *P* = .9668), or ODI scale (slope coefficient = −0.0040 (0.0142), *P* = .7801).

#### 3.7.3. Dosage of cupping.

Details on Dosage were available in 5 studies (availability of information: 235/460 patients, 51.1%). Meta-regression showed no statistically significant association between the dosage of cupping and the ODI scale (slope coefficient = −0.2004 (0.1093), *P* = .0668).

## 4. Discussion

Cupping treatments for LBP, such as dry cupping or wet cupping, resulted in different pain reduction effects, as predicted. However, both dry and wet cupping can be used to improve the quality of life of LBP patients.

The VAS and PPI are the scales administered to quantify and record subjective pain intensity.^[22,29]^ There is a strong correlation between VAS, PPI scores, and the pain level of patients with LBP.^[57,58]^ LBP patients often use VAS and PPI pain ratings to measure their pain.^[59]^ And pain ratings can be used to demonstrate clinically significant benefits of the therapeutic effect of treatment in LBP.^[48]^

In our results, wet cupping reduces the scores of PPI. However, While the dry cupping group showed a reduction in VAS after treatment, the effect was not significantly different from the control group in the forest plot. The finding was not similar to the past study, which is in that cupping significantly decreased pain symptoms.^[28,57,58]^ A classification of cupping types or a score based on self-management could explain this.

Surprisingly, this illustrates the difference in the effectiveness of dry and wet cupping in the reduction of pain. Surprisingly, this illustrates the difference in the effectiveness of dry and wet cupping in quality of life. The most significant difference between dry cupping and wet cupping is wet cupping procedure generally involves lacerating the skin and extracting a small amount of blood.^[54]^ The wet cupping therapy can improve the health-related quality of life by cleansing the body from morbid humor by extracting blood.^[60]^ This may be similar to the bloodletting treatment that can divert the morbid substances from the vital organs like the brain, heart, and liver to evacuees the humor to the body and reestablishing the body’s robustness.^[61]^ Furthermore, the mechanism of wet cupping treatments is confirmed could reduce the symptoms of diseases such as endocrine^[57]^ by eliminating cholesterol, free radicals, and heavy metals which are destructive or harmful substances from the body.^[36,61]^

The cholesterol, free radicals, and heavy metals can affect wet cupping therapy with LBP. In the impact of cholesterol. According to studies, severe pain caused by neurogenic symptoms may be associated with higher cholesterol levels.^[62]^ Additionally, cholesterol may exacerbate LBP caused by atherosclerosis.^[63]^ In the effect of free radicals, endorphins, genes-related peptides, and steroids may also contribute to LBP, such as inflammatory or neuropathic pain.^[64]^ Pain modulation is mainly associated with these compounds.^[65]^ The effect of heavy metals, such as Cadmium, has been shown to damage kidneys and cause back pain.^[1]^

And as we know, both the PPI and VAS are reliable pain scales, but their applications are different. The VAS visualizes pain,^[39]^ while the PPI describes the pain as a degree of pain.^[40]^ Furthermore, VAS was found to be highly correlated with pretreatment pain levels, whereas PPIs were not.^[66]^ The effectiveness of dry cupping in reducing pain may have varied between the two.

The effect on ODI in our result includes 4 studies of wet cupping^[21,37,53,54]^ and 2 studies of dry cupping.^[23,55]^ The ODI is a comprehensive assessment tool designed for assessing disability related to back pain and function, which measures the impact of back pain on quality of life.^[23]^ ODI scales showed both the dry and wet cupping experiment group’s self-management scale is lower than the control group, suggesting moist cupping therapy can improve physical and social functions associated with LBP.^[49]^

We noted that these studies indicated that both dry and wet cupping groups did achieve the effect of lowering LBP to improve the patients’ sentiments. In addition, our finding was similar to the study of Seo et al^[56]^ in that cupping significantly decreased pain symptoms. We demonstrate further by classification that both modalities can be ameliorating the quality of life. It is negative pressure that is common to both dry and wet cupping that may cause these effects. Cupping therapy may reduce LWP by activating a negative pressure microenvironment.^[32]^ It has been suggested that the concepts of pain-gate and conditioned pain modulation describe the biological and mechanical basis for pain relief associated with negative pressure therapy for dry and wet cupping therapy.^[58]^ According to pain-gate, negative pressure suppresses pain through mechanoreceptor stimulation of nerve impulses that “close the gates”^[67]^ Furthermore, conditioned pain suggested that cupping therapy stimulation the skin with several autonomous, hormonal, and immune reactions, activating the neuroendocrine-immune system and reducing pain.^[30]^

This study’s heterogeneity for *I*^*2*^ of the PPI, and ODI scales was higher than 50%. Therefore, they were considered to indicate high heterogeneity. Since there was significant heterogeneity, we conducted meta-regression analyses to find the reason. After analysis, the heterogeneity remained very high despite the same intervention (dry, wet cupping) and the same evaluation factors (VAS, PPI, and ODI).

There has never been a definitive formula for specific plans for cupping therapy, as far as we know. Meta-analyses of multi-team studies conducted in different locations and using different methods are characterized by heterogeneity. Since heterogeneity is inevitable in the analysis, the critical question arises regarding what triggered the heterogeneity.^[66]^ Although cupping treatments were found to provide the same benefits in most studies (e.g., pain relief or a reduction in ODI), there was still significant heterogeneity across the studies. To detect differences that may cause high heterogeneity, we will perform regression analyses on the articles. Regression analysis showed that the number of treatment times had a significant effect on the outcome intercept after treatment, explaining the high heterogeneity between studies.

This present review has several limitations. In the first limitation, as we know, no studies were conducted on a trial study comparing wet cupping with dry cupping. Thus, it is not easy to distinguish the therapeutic differences between the 2 therapies. Therefore, this paper can only analyze the effect through the quantitative analysis of different experiments. The consistency of the results was also affected by the number of treatment times across studies. In the second limitation, the included RCTs had methodological limitations that may have induced bias in the results. The methodological limitations’ main manifestation of this is the difficulty in implementing blind tests. Our research showed that cupping therapy might be a treatment option for LBP, but the clinical heterogeneity and risk of bias still limit the evidence.^[45]^ In the third limitation, this study focused on papers published in English. However, studies published in other languages may affect the results. 83 Cupping therapy has been popular in most countries^[35,54,68]^ Therefore, more research in publishing with no restriction on language and publication type is required.^[69]^

## 5. Conclusions

This review highlights dry and wet cupping therapy in managing pain and disability for LBP. Our study’s originality is that it analyses wet and dry cupping samples to determine the effectiveness of pain self-management in LBP.

In the present meta-analysis, it is demonstrated that cupping therapy interventions may be beneficial for the treatment of LBP. There is no evidence that dry cupping reduces the pain associated with LBP, but it does seem to improve the quality of life. In contrast, wet cupping reduced pain intensity and improved the quality of life in people with LBP.

## Acknowledgments

The authors wish to express gratitude to Mr. Fahni Haris, Ms. Yori Pusparani, and Mr. Jifeng Wang for their assistance.

## Author contributions

All authors have read and agreed to the published version of the manuscript.

**Conceptualization:** Wei-Cheng Shen, Chi-Wen Lung.

**Methodology:** Song Wang, Chien-Cheng Tai.

**Supervision:** Yih-Kuen Jan, Ben-Yi Liau.

**Writing – original draft:** Wei-Cheng Shen, Quanxin Lin.

**Writing – review and editing:** Yih-Kuen Jan, Chi-Wen Lung.

## Supplementary Material

## References

[1] Zhang Y Liang Z Li S. Fire needle plus cupping for acute herpes zoster: study protocol for a randomized controlled trial. Trials. 2020;21:701. 32762718 10.1186/s13063-020-04599-2 PMC7409425

[2] Cunha LL Mayrink WC. Influence of chronic pain in the quality of life of the elderly. Revista Dor. 2011;12:120–4.

[3] George SZ Goertz C Hastings SN. Transforming low back pain care delivery in the United States. Pain. 2020;161:2667–73. 32694378 10.1097/j.pain.0000000000001989 PMC7669560

[4] Manchikanti L Singh V Falco FJ. Epidemiology of low back pain in adults. Neuromodulation. 2014;17(Suppl 2):3–10. 25395111 10.1111/ner.12018

[5] Pergolizzi JV Jr LeQuang JA. Rehabilitation for low back pain: a narrative review for managing pain and improving function in acute and chronic conditions. Pain Ther. 2020;9:83–96. 32006236 10.1007/s40122-020-00149-5 PMC7203283

[6] Cashin AG Rizzo RRN Wand BM. Non-pharmacological and non-surgical treatments for low back pain in adults: an overview of Cochrane reviews. Cochrane Database Syst Rev. 2021;2021:10.1002/14651858.CD014691. 10.1002/14651858.CD013815.pub2 PMC10072849 37014979

[7] Piano L Ritorto V Vigna I. Individual patient education for managing acute and/or subacute low back pain: little additional benefit for pain and function compared to placebo. A systematic review with meta-analysis of randomized controlled trials. J Orthop Sports Phys Ther. 2022;52:432–45. 35584025 10.2519/jospt.2022.10698

[8] Teut M Kaiser S Ortiz M. Pulsatile dry cupping in patients with osteoarthritis of the knee – a randomized controlled exploratory trial. BMC Complement Altern Med. 2012;12:184. 23057611 10.1186/1472-6882-12-184 PMC3527288

[9] Ma K Zhuang ZG Wang L. The Chinese Association for the Study of Pain (CASP): consensus on the assessment and management of chronic nonspecific low back pain. Pain Res Manag. 2019;2019:8957847. 31511784 10.1155/2019/8957847 PMC6714323

[10] Bindu S Mazumder S Bandyopadhyay U. Non-steroidal anti-inflammatory drugs (NSAIDs) and organ damage: a current perspective. Biochem Pharmacol. 2020;180:114147. 32653589 10.1016/j.bcp.2020.114147 PMC7347500

[11] Park AL Hwang EH Hwang MS. Cost-effectiveness of Chuna manual therapy and usual care, compared with usual care only for people with neck pain following traffic accidents: a multicenter randomized controlled trial. Int J Environ Res Public Health. 2021;18:9994. 34639295 10.3390/ijerph18199994 PMC8508460

[12] Fairbank J Frost H Wilson-MacDonald J. Randomised controlled trial to compare surgical stabilisation of the lumbar spine with an intensive rehabilitation programme for patients with chronic low back pain: the MRC spine stabilisation trial. BMJ. 2005;330:1233. 15911537 10.1136/bmj.38441.620417.8F PMC558090

[13] Foster NE Anema JR Cherkin D Lancet Low Back Pain Series Working Group. Prevention and treatment of low back pain: evidence, challenges, and promising directions. Lancet. 2018;391:2368–83. 29573872 10.1016/S0140-6736(18)30489-6

[14] Baliga S Treon K Craig NJ. Low back pain: current surgical approaches. Asian Spine J. 2015;9:645–57. 26240729 10.4184/asj.2015.9.4.645 PMC4522460

[15] Liu L Tang Y Baxter GD. Complementary and alternative medicine – practice, attitudes, and knowledge among healthcare professionals in New Zealand: an integrative review. BMC Complement Med Ther. 2021;21:63. 33583417 10.1186/s12906-021-03235-z PMC7882070

[16] Teut M Ullmann A Ortiz M. Pulsatile dry cupping in chronic low back pain – a randomized three-armed controlled clinical trial. BMC Complement Altern Med. 2018;18:115. 29609566 10.1186/s12906-018-2187-8 PMC5879872

[17] Silva HJA Saragiotto BT Silva RS. Dry cupping in the treatment of individuals with non-specific chronic low back pain: a protocol for a placebo-controlled, randomised, double-blind study. BMJ Open. 2019;9:e032416. 10.1136/bmjopen-2019-032416 PMC6937004 31871257

[18] Wang D. Effect of cupping therapy in the treatment of low back pain among nurses in China. J Altern Complement Med. 2020;6:1–4.

[19] Lin ML Wu HC Hsieh YH. Evaluation of the effect of laser acupuncture and cupping with Ryodoraku and visual analog scale on low back pain. Evid Based Complement Alternat Med. 2012;2012:521612. 23118792 10.1155/2012/521612 PMC3482015

[20] Lin ML Wu JH Lin CW. Clinical effects of Laser acupuncture plus Chinese cupping on the pain and plasma cortisol levels in patients with chronic nonspecific lower back pain: a randomized controlled trial. Evid Based Complement Alternat Med. 2017;2017:3140403. 28848615 10.1155/2017/3140403 PMC5564089

[21] AlBedah A Khalil M Elolemy A. The use of wet cupping for persistent nonspecific low back pain: randomized controlled clinical trial. J Altern Complement Med. 2015;21:504–8. 26069973 10.1089/acm.2015.0065 PMC4522952

[22] Magri LV Carvalho VA Rodrigues FC. Effectiveness of low-level laser therapy on pain intensity, pressure pain threshold, and SF-MPQ indexes of women with myofascial pain. Lasers Med Sci. 2017;32:419–28. 28054261 10.1007/s10103-016-2138-x

[23] Razali AI Choo LA. The effectiveness of dry cupping and hot pack in pain relief and reduce functional disability on non-specific low back pain. Eur J Mol Clin Med. 2021;8:2796–810.

[24] Wood S Fryer G Tan LLF. Dry cupping for musculoskeletal pain and range of motion: a systematic review and meta-analysis. J Bodyw Mov Ther. 2020;24:503–18. 33218554 10.1016/j.jbmt.2020.06.024

[25] Lauche R Cramer H Hohmann C. The effect of traditional cupping on pain and mechanical thresholds in patients with chronic nonspecific neck pain: a randomised controlled pilot study. Evid Based Complement Alternat Med. 2012;2012:429718. 22203873 10.1155/2012/429718 PMC3235710

[26] Moura CC Chaves C Cardoso A. Cupping therapy and chronic back pain: systematic review and meta-analysis. Rev Lat Am Enfermagem. 2018;26:e3094. 30462793 10.1590/1518-8345.2888.3094 PMC6248735

[27] Mueller C. The effects of massage therapy on delayed onset muscle soreness. University of Northern Iowa, Cedar Falls. 2018:671.

[28] Lee MS Kim JI Ernst E. Is cupping an effective treatment? An overview of systematic reviews. J Acupunct Meridian Stud. 2011;4:1–4. 21440874 10.1016/S2005-2901(11)60001-0

[29] Wang YT Qi Y Tang FY. The effect of cupping therapy for low back pain: a meta-analysis based on existing randomized controlled trials. J Back Musculoskelet Rehabil. 2017;30:1187–95. 28946531 10.3233/BMR-169736

[30] Aboushanab TS AlSanad S. Cupping therapy: an overview from a modern medicine perspective. J Acupunct Meridian Stud. 2018;11:83–7. 29436369 10.1016/j.jams.2018.02.001

[31] Soliman Y Hamed N Khachemoune A. Cupping in dermatology: a critical review and update. Acta Dermatovenerol Alp Pannonica Adriat. 2018;27:103–7. 29945267

[32] Wang SZ Lu YH Wu M. Cupping therapy for diseases: an overview of scientific evidence from 2009 to 2019. Chin J Integr Med. 2021;27:394–400. 32524396 10.1007/s11655-020-3060-y

[33] Mehta P Dhapte V. Cupping therapy: a prudent remedy for a plethora of medical ailments. J Tradit Complement Med. 2015;5:127–34. 26151023 10.1016/j.jtcme.2014.11.036 PMC4488563

[34] Du S Hu L Dong J. Self-management program for chronic low back pain: a systematic review and meta-analysis. Patient Educ Couns. 2017;100:37–49. 27554077 10.1016/j.pec.2016.07.029

[35] Mardani-Kivi M Montazar R Azizkhani M. Wet-cupping is effective on persistent nonspecific low back pain: a randomized clinical trial. Chin J Integr Med. 2019;25:502–6. 30484021 10.1007/s11655-018-2996-0

[36] Joushan A Choopani R Agin K. The role of manual therapy/practices (dry cupping, wet cupping, leech therapy, venesection, or phlebotomy) in lung diseases. Eur J Mol Clin Med. 2020;7:4444–54.

[37] Al-Eidi SM Mohamed AG Abutalib RA. Wet cupping-traditional Hijamah technique versus Asian cupping technique in chronic low back pain patients: a pilot randomized clinical trial. J Acupunct Meridian Stud. 2019;12:173–81. 31028971 10.1016/j.jams.2019.04.005

[38] Garratt A Schmidt L Mackintosh A. Quality of life measurement: bibliographic study of patient assessed health outcome measures. BMJ. 2002;324:1417. 12065262 10.1136/bmj.324.7351.1417 PMC115850

[39] Kitisomprayoonkul W Klaphajone J Kovindha A. Thai short-form McGill pain questionnaire. J Med Assoc Thai. 2006;89:846–53. 16850687

[40] Ho K Spence J Murphy MF. Review of pain-measurement tools. Ann Emerg Med. 1996;27:427–32. 8604852 10.1016/s0196-0644(96)70223-8

[41] Brett J Staniszewska S Mockford C. A systematic review of the impact of patient and public involvement on service users, researchers and communities. Patient. 2014;7:387–95. 25034612 10.1007/s40271-014-0065-0

[42] Monticone M Baiardi P Vanti C. Responsiveness of the Oswestry disability index and the Roland Morris disability questionnaire in Italian subjects with sub-acute and chronic low back pain. Eur Spine J. 2012;21:122–9. 21823035 10.1007/s00586-011-1959-3 PMC3252446

[43] Dobrescu AI Nussbaumer-Streit B Klerings I. Restricting evidence syntheses of interventions to English-language publications is a viable methodological shortcut for most medical topics: a systematic review. J Clin Epidemiol. 2021;137:209–17. 33933579 10.1016/j.jclinepi.2021.04.012

[44] Kim S Lee SH Kim MR. Is cupping therapy effective in patients with neck pain? A systematic review and meta-analysis. BMJ Open. 2018;8:e021070. 10.1136/bmjopen-2017-021070 PMC6231582 30397006

[45] Cramer H Klose P Teut M. Cupping for patients with chronic pain: a systematic review and meta-analysis. J Pain. 2020;21:943–56. 31982686 10.1016/j.jpain.2020.01.002

[46] Moher D Liberati A Tetzlaff J PRISMA Group. Preferred reporting items for systematic reviews and meta-analyses: the PRISMA statement. PLoS Med. 2009;6:e1000097. 19621072 10.1371/journal.pmed.1000097 PMC2707599

[47] Jensen MP Nielson WR Kerns RD. Toward the development of a motivational model of pain self-management. J Pain. 2003;4:477–92. 14636816 10.1016/s1526-5900(03)00779-x

[48] Oliveira VC Ferreira PH Maher CG. Effectiveness of self-management of low back pain: systematic review with meta-analysis. Arthritis Care Res. 2012;64:1739–48. 10.1002/acr.21737 22623349

[49] Koç M Bayar B Bayar K. A comparison of back pain functional scale with roland morris disability questionnaire, Oswestry disability index, and short form 36-health survey. Spine. 2018;43:877–82. 28984734 10.1097/BRS.0000000000002431

[50] Huedo-Medina TB Sánchez-Meca J Marín-Martínez F. Assessing heterogeneity in meta-analysis: Q statistic or I^2^ index? Psychol Methods. 2006;11:193–206. 16784338 10.1037/1082-989X.11.2.193

[51] Kontopantelis E Reeves D. Performance of statistical methods for meta-analysis when true study effects are non-normally distributed: a simulation study. Stat Methods Med Res. 2012;21:409–26. 21148194 10.1177/0962280210392008

[52] Hoy D Brooks P Woolf A. Assessing risk of bias in prevalence studies: modification of an existing tool and evidence of interrater agreement. J Clin Epidemiol. 2012;65:934–9. 22742910 10.1016/j.jclinepi.2011.11.014

[53] Farhadi K Schwebel DC Saeb M. The effectiveness of wet-cupping for nonspecific low back pain in Iran: a randomized controlled trial. Complement Ther Med. 2009;17:9–15. 19114223 10.1016/j.ctim.2008.05.003

[54] Kim J-I Kim T-H Lee MS. Evaluation of wet-cupping therapy for persistent non-specific low back pain: a randomised, waiting-list controlled, open-label, parallel-group pilot trial. Trials. 2011;12:146–52. 21663617 10.1186/1745-6215-12-146 PMC3141528

[55] Silva HJA Barbosa GM Silva RS. Dry cupping therapy is not superior to sham cupping to improve clinical outcomes in people with non-specific chronic low back pain: a randomized trial. J Physiother. 2021;67:132–9. 33757719 10.1016/j.jphys.2021.02.013

[56] Seo J Chu H Kim CH. Cupping therapy for migraine: a PRISMA-compliant systematic review and meta-analysis of randomized controlled trials. Evid-based Complement Altern Med. 2021;2021:1–9. 10.1155/2021/7582581 PMC8016589 33833822

[57] Al-Shidhani A Al-Mahrezi A; Novel Therapies, Bioactives. The role of cupping therapy in pain management: a literature. J AYUSH Res. 2021;1:217.

[58] Chen L Ferreira ML Beckenkamp PR. Comparative efficacy and safety of conservative care for pregnancy-related low back pain: a systematic review and network meta-analysis. Phys Ther. 2021;101:pzaa200. 33210717 10.1093/ptj/pzaa200

[59] Edwards K Rosenthal B O’Connor JJ. Long term efficacy of botulinum toxin type A for post laminectomy syndrome. J Pain. 2007;8:S38.

[60] Ersoy S Habibe I Sunay D. Wet cupping therapy improves health related quality of life: a self-controlled interventional study. Ankara Med J. 2019;19:270–7.

[61] Nimrouzi M Mahbodi A Jaladat AM. Hijamat in traditional Persian medicine: risks and benefits. J Evid Based Complementary Altern Med. 2014;19:128–36. 24647093 10.1177/2156587214524578

[62] Kauppila LI McAlindon T Evans S. Disc degeneration/back pain and calcification of the abdominal aorta. A 25-year follow-up study in Framingham. Spine. 1997;22:1642–7; discussion 1648. 9253101 10.1097/00007632-199707150-00023

[63] Kauppila LI. Atherosclerosis and disc degeneration/low-back pain – a systematic review. Eur J Vasc Endovasc Surg. 2009;37:661–70. 19328027 10.1016/j.ejvs.2009.02.006

[64] Mensah-Nyagan AG Meyer L Schaeffer V. Evidence for a key role of steroids in the modulation of pain. Psychoneuroendocrinology. 2009;34(Suppl 1):S169–77. 19577851 10.1016/j.psyneuen.2009.06.004

[65] de Mos M Sturkenboom MC Huygen FJ. Current understandings on complex regional pain syndrome. Pain Pract. 2009;9:86–99. 19215592 10.1111/j.1533-2500.2009.00262.x

[66] Adelmanesh F Jalali A Attarian H. Reliability, validity, and sensitivity measures of expanded and revised version of the short-form McGill Pain Questionnaire (SF-MPQ-2) in Iranian patients with neuropathic and non-neuropathic pain. 2012;13:1631–8. 10.1111/j.1526-4637.2012.01517.x 23137190

[67] Kouser HV Nayab M Tehseen A. Evidence-based therapeutic benefits of cupping therapy (Hijāma): a comprehensive review. J Drug Deliv Ther. 2021;11:258–62.

[68] Cao H Han M Li X. Clinical research evidence of cupping therapy in China: a systematic literature review. BMC Complement Altern Med. 2010;10:70. 21078197 10.1186/1472-6882-10-70 PMC3000376

[69] Cao H Li X Liu J. An updated review of the efficacy of cupping therapy. PLoS One. 2012;7:e31793. 22389674 10.1371/journal.pone.0031793 PMC3289625

