# Supplementary material for: Effectiveness of self-management of dry and wet cupping therapy for low back pain: A systematic review and meta-analysis
Source: Medicine (Baltimore). 2022 Dec 23;101(51):e32325. doi: 10.1097/MD.0000000000032325 (PMC9794267; doi:10.1097/MD.0000000000032325)
Supplement: Supplementary file 1 [file medi-101-e32325-s001.pdf]

Database	Algorithm
PubMed	(cupping therapy or bleeding cupping or wet cupping) AND (Low back pain or cupping therapy pain)
Web of Science	((((ALL=(Cupping therapy)) OR ALL=(bleeding cupping)) OR ALL=(wet cupping)) AND ALL=(Low back pain)) AND ALL=(cupping therapy pain)
Cochrane Library	(cupping therapy or bleeding cupping or wet cupping) AND (Low back pain or cupping therapy pain)
Scopus	(cupping therapy or bleeding cupping or wet cupping) AND (Low back pain or cupping therapy pain)
CINAHL	(cupping therapy or bleeding cupping or wet cupping) AND (Low back pain or cupping therapy pain)
